# Variability in triggers for mechanical left ventricular unloading in VA-ECMO: A literature search

**DOI:** 10.1051/ject/2024031

**Published:** 2025-03-07

**Authors:** Anthony Calhoun, Min-Ho Lee, Dominic V. Pisano, Alexandros Karavas, Jamel Ortoleva

**Affiliations:** 1 Department of Perfusion, Boston Medical Center 732 Harrison Ave 3rd Floor Boston MA 02118 USA; 2 Perfusion Services, Children’s Hospital of Philadelphia 3401 Civic Center Blvd Philadelphia PA 19104 USA; 3 Department of Anesthesiology, Boston Medical Center 750 Albany Street, Floor 2R, Power Plant Building Boston MA 02118 USA; 4 Division of Cardiac Surgery, Boston Medical Center 750 Albany Street Boston MA 02118 USA

**Keywords:** Extracorporeal membrane oxygenation (ECMO), Left ventricular unloading, Left ventricular distension, Mechanical unloading, Literature review, Venoarterial ECMO (V-A ECMO)

## Abstract

*Background*: Venoarterial Extracorporeal Membrane Oxygenation (VA-ECMO) is a means of supporting the lungs or the heart and lungs in patients with hemodynamic compromise that is refractory to conventional measures. VA-ECMO is most commonly deployed in a percutaneous fashion with femoral arterial and venous access. While VA-ECMO, particularly in a femoral-femoral configuration, provides both hemodynamic and ventilatory support, it also causes increased afterload on the left ventricle (LV) which in turn may result in LV distension (LVD). LV thrombus formation, ventricular arrhythmias, pulmonary edema, and pulmonary hemorrhage are clinical manifestations of LVD. LV unloading is a means of preventing LVD and its sequelae. If less invasive methods fail to achieve adequate LV unloading, invasive mechanical methods are pursued such as intra-aortic balloon pump counter-pulsation, atrial septostomy, surgical venting, left atrial cannulation, and percutaneous transvalvular micro-axial pump placement. *Methods*: We sought to review indicators of LVD, thresholds, and options for mechanical venting strategies. A Pubmed search was performed to identify current literature about LV unloading for VA ECMO. This was categorized and summarized to determine commonly reported thresholds for mechanical LV unloading. *Results*: Multiple physiologic and radiographic indicators were reported without uniformity. Common indicators included increased pulmonary artery catheter pressures, decreased Aortic Line Pulse Pressure, as well as multiple Echocardiographic, and radiographic indicators. *Conclusion*: Although there has been significant interest in the topic, there is currently limited uniformity in thresholds for when to initiate and escalate mechanical LV unloading. While the method of LV unloading is an active area of investigation, the threshold for which to initiate invasive venting strategies is largely unexplored.

## Introduction

Venoarterial Extracorporeal Membrane Oxygenation (VA-ECMO) increases left ventricle (LV) afterload and in certain instances reduces aortic valve (AV) opening resulting in LV distension (LVD) [1–3]. Unloading the LV during VA-ECMO is an important maneuver to treat and avoid complications such as pulmonary edema, pulmonary hemorrhage (if due to elevated left atrial pressure), ventricular arrhythmias, LV thrombus burden, and aortic root thrombosis caused by LVD and lack of AV opening [2, 4]. LV unloading can be accomplished either by non-invasive maneuvers such as afterload reduction (for example, using vasodilators, decreasing VA-ECMO flow, increasing positive end-expiratory pressure), inotropic support, and diuresis, or by invasive mechanical methods [2, 5]. Invasive mechanical methods are effective but come with an increased risk of access site complications, hemolysis, and other drawbacks [6, 7]. A growing body of randomized and retrospective literature exists regarding the effects of LV unloading on outcomes [7–12]. Despite ongoing research on the use of unloading strategies, the optimal strategy of monitoring, triggers, and methods of mechanical unloading in VA-ECMO have not been established. At the current time, there is no standard of care escalation pathway for mechanical unloading in the VA-ECMO population, though guidelines do exist based on clinical, hemodynamic, and radiographic evidence of pulmonary congestion [5]. Unfortunately, these guidelines are not supported by randomized evidence and the expanding options for LV mechanical unloading significantly complicate decision-making and add inter-institutional variability to this process due to varying experience with individual technologies. In addition to physiologic differences in the mechanical methods of LV unloading, placement techniques for these strategies vary significantly. For example, atrial septostomy requires expertise in structural heart interventions while intra-aortic balloon pump counter pulsation and percutaneous transvalvular micro-axial pump placement require a separate skill set. While some studies suggest an association between improved hospital mortality with mechanical LV unloading during VA-ECMO, long-term outcomes are less clear [7]. One small study of cardiogenic shock patients suggested an association with improved 90-day survival in those supported by VA-ECMO (not due to myocardial infarction-related shock) undergoing concomitant IABP [13]. For example, a recent study from the United Network for Organ Sharing (UNOS) on patients proceeding to transplant did not suggest a difference in survival in VA-ECMO patients undergoing LV mechanical unloading versus those supported by VA-ECMO without it [14].

LVD is important for patients with cardiogenic shock as it may negatively impact cardiac recovery. Early recognition and management may improve outcomes. LVD may occur upon (or even prior to) initiation of VA-ECMO support and methods of detection are based on clinical, hemodynamic, or imaging information. Depending on institutional preferences, pulmonary artery catheter (PAC) hemodynamics, echocardiographic (ECHO) evaluation, arterial line pulse pressure (ALPP) assessment, and the presence of pulmonary congestion on chest radiography have been suggested as assessment tools for the detection of LV distention and its sequelae [1, 2, 5, 6].

Clinical trials, review articles and editorials have described multiple indications for the use of LV unloading and display significant heterogeneity in triggers for the initiation of this maneuver [15]. A recent survey of Italian medical centers showed significant practice variation in both monitoring and treatment modalities for LV unloading in patients supported by VA-ECMO [16]. Additionally, certain clinical trials were designed to initiate mechanical LV unloading at the time of VA-ECMO initiation for cardiogenic shock [8] (See Table 1). This variability makes research more challenging to interpret and increases the complexity of clinical decision-making.

**Table 1:** Literature survey of Clinical, Hemodynamic, and Echocardiographic indications of LV distension that potentially trigger the mechanical LV unloading.

Author, Publication Date, Type, Ref #	Hemodynamic indications	Echocardiographic indications	Recommended LV unloading
Truby et al., 2017, RA, [1]	PAD > 25	LV blood stasis	TVMA
Ezad et al., 2023, RA, [2]	PCWP > 18, or ALPP < 15	Increased LV dimensions, LV blood stasis, LV thrombus, No AV opening, LVOT VTI < 10 cm	IABP, TVMA
Cevasco et al., 2019 RA, [3]	PAD > 25; “an elevated PCWP”	LV distension, LV blood stasis, LV thrombus, hypocontractile LV, No AV Opening	TVMA, Surg Vent
Lorusso et al., 2021 GD, [5]	Moderate: CVP 12–16, PCWP 18–25 (moderate); Severe: CVP Above 20, PCWP above 25	Moderate: AV opening every 3–4 beats, moderate LV/LA distension, moderate Echo Smoke, IVC over 2.5 cm dilated, IVC collapse less than 50%; Severe: AV closed, Severe LV/LA distention, Severe Echo Smoke, IVC over 2.5 cm, no IVC collapse.	IABP, AS, Surg Vent, TVMA
Kim et al., 2023, RT, [8]	Minimal ALPP	LV Blood Stasis, No AV Opening, Low ALPP	TSLAV
Park et al., 2023, RT, [9]	No Hemodynamic Criteria Described	No or Low AV Opening, congestion score index	TSLAV
Cheng et al., 2013, CS, [10]	PCWP > 18	EF < 20%, Low or No AV Opening, LV Distension, LV Blood Stasis, Echo Smoke	TVMA
Hasde et al., 2021, RR, [12]	PAD > 25, PCWP at least 20	Low or No AV opening	IABP, AS, Surg Vent
Thiele et al., 2023, RT, [17]	Lack of ALPP	No AV opening, increase in diameters and volume of LV, LVOT VTI < 10 cm	IABP, TVMA
Assmann et al., 2022, GD, [18]	PAD > 25	LV Dilation	IABP, AS, Surg Vent, TVMA
Belohlavek et al., 2021, RA, [19]	ALPP < 15, high LVEDP	High LV Filling Pressures by Doppler Echocardiography	TVMA
Donker et al., 2022, Ed, [20]	Increased PAC Pressures; Reduced ALPP	Echo Smoke, Low or No AV opening	TVMA
Gaisendrees et al., 2021, RR, [21]	Low ALPP	Echo Smoke, LVEDD at Least 6.8 cm (male), 6.1 (female)	TVMA
Lim et al., 2021, RA, [22]	Rising PAP and PCWP, Reduced ALPP	LV Dilation, Echo Smoke, Low or No AV opening	TVMA
Lorusso et al., 2022, RR, [23]	CVP 12–16 (moderate), above 20 (severe); ALPP: 8–10 (moderate), less than 8 or pulseless (severe); wedge (PCWP?): 20–25 (moderate), above 25 (severe), Scvo2: 55–45 (moderate), under 45 (severe)	LA/LV distension, Echo Smoke, IVC: 1.5–2.5, above 2.5 for mild, moderate/severe	IABP, TVMA, Surg Vent
Lüsebrink et al., 2023, RA, [24]	No ALPP, Elevated PAP or PCWP	Closed AV, LV Blood Stasis	Multiple Discussed
Meani et al., 2019, RR, [25]	Moderate PCWP 18–25, CVP 12–16; Severe: CVP > 20, PCWP > 25; Low or No ALPP	Moderate: AV opening every 3–4 beats, moderate LV/LA distension, moderate smoke like effect, IVC over 2.5 cm dilated, IVC collapse less than 50%; Severe: AV closed, LV/LA distention, Severe smoke like effect, IVC over 2.5 cm, no IVC collapse	IABP, TVMA
Nakajima et al., 2021, RR, [26]	ALPP < 20	Echo Smoke	TVMA
Piechura et al., 2020, RR, [27]	ALPP < 10	LV Dilation or Low or no AV opening	IABP, TVMA
Ricarte Bratti et al., 2021, RA, [28]	Elevated LV Filling Pressures, ALPP < 10	Increased LVEDD, increased E/E’ ratio, Echo Smoke, LV Thrombus, Low or No AV Opening	IABP, AS, TVMA, Surg Vent
Alkhouli et al., 2016, CS, [29]	PCWP > 18, “High Left Atrial Pressure”	No Echocardiographic indications were noted	Surgical Vent, AS, TVMA
Au et al., 2023, RR, [30]	No Hemodynamic Criteria Described	LVEF < 25%	IABP, TVMA
Eliet et al., 2018, RR, [31]	ALPP < 10	No AV opening, heavy Echo Smoke in LV, LVOT VTI < 5 cm	TVMA
Gaudard et al., 2015, RR, [32]	No Hemodynamic Criteria Described	Acute LV dilation/ Echo Smoke in LV/LA	TVMA
Hu et al., 2016, CS, [33]	Decreased ALPP	LV Blood Stasis	IABP
Karatolios et al., 2016, RR, [34]	No Hemodynamic Criteria Described	Echo Smoke in LV, LV Dilation, Low or No AV opening	TVMA
Kim et al., 2021 RR, [35]	ALPP < 10	No Echocardiographic indications were noted	TVMA
Lüsebrink et al., 2020 RA, [36]	Lack of ALPP	Low or No AV opening, LVOT VTI < 10 cm, LV Blood Stasis, Increased LV Dimensions from Previous Exam, severe AR	TVMA
Pappalardo et al., 2017, RR, [37]	No Hemodynamic Criteria Described	Stone Heart, LV Thrombus, significant AR	TVMA
Rali et al., 2022, RA, [38]	elevated PCWP, low or absent ALPP	No AV opening	IABP, TVMA, AS
Saeed et al., 2023, RA, [39]	ALPP < 15, PCWP > 30, PAD > 25	LV/ Ao Root Thrombus, No AV opening	IABP, TVMA, AS, TSLAV, LV Vent

Thresholds for defining LVD and indications and triggers for LV mechanical unloading were tabulated according to categories of clinical (or radiographic), hemodynamic, and ECHO findings or parameters. Clinical and radiologic criteria for LVD were placed within the same category for simplicity and to be succinct. Hemodynamic manifestations of LVD were defined as abnormalities with invasive filling pressures and ALPP monitoring. All pressures are reported in mmHg. ECHO criteria for LVD included cardiac ultrasound or pulmonary findings suggestive of pulmonary edema. Several papers stratified their indications and treatments as mild, moderate, and severe. Where applicable this has been included. ALPP: Arterial Line Pulse Pressure; AV: Aortic Valve; AS: Atrial Septostomy; CS: Case Series; CVP: Central Venous Pressure; Ed: Editorial; GD: Guideline Document; IABP: Intra Aortic Balloon Pump; IVC: Inferior Vena Cava; LA: Left Atrium; LV: Left Ventricle; LVD: Left Ventricular Distension; LVOT VTI: Left Ventricular Outflow Tract Velocity Time Integral; PA: Pulmonary Artery; PAC: Pulmonary Artery Catheter; PAD: Pulmonary Artery Diastolic; PCWP: Pulmonary Capillary Wedge Pressure; RA: Review Article; RR: Retrospective Review; RT: Randomized Trial; TSLAV: Transeptal Left Atrial Vent.

Not all LV unloading strategies are equivalent. As the clinician increases the level of invasiveness in unloading, risks, and negative sequelae increase for the patient. The lack of uniformity in the literature makes the indications of care escalation unclear as well. Additionally, in cases where the AV is not opening due to acute hypovolemia, invasive methods of LV unloading are not only unwarranted but will likely be ineffective. This literature search serves to call attention to further investigation to answer the question of when and how to unload the LV in the setting of VA-ECMO.

## Methods

A PubMed search was performed using the phrase “LEFT VENTRICULAR UNLOADING ECMO” to retrieve all research, review, and editorial articles that resulted under this search term. The articles were reviewed for suggested criteria to diagnose LVD and thresholds for LV mechanical unloading. Articles were separated by type (for example, research (randomized or otherwise), guidelines, review, and editorial). In this literature search, we identified both qualitative and quantitative criteria that were clinical (and radiological), hemodynamic, and echocardiographic in nature. Articles were excluded from this review if they did not describe criteria for LVD or thresholds for mechanical LV unloading. For example, articles that described outcomes associated with LV mechanical unloading but did not describe thresholds were not included in this review.

## Summary of literature search

Significant heterogeneity was noted in definitions of LVD and triggers for the initiation of LV mechanical unloading. We found that significant variability in the diagnosis and treatment of LVD during VA-ECMO exists, which is consistent with a recent countrywide survey of Italian centers [16].

Three randomized trials either examine the effect of mechanical unloading or contain a threshold for its initiation [8, 9, 17]. Of these three, two studies examine the effects of transseptal cannulation for left atrial drainage to mechanically unload the LV, both of which did not find an impact on survival [8, 9]. The main drawbacks of these studies were their small size and use of a relatively uncommon, and indirect method of LV unloading (specifically, left atrial drainage that reduces LV preload as opposed to direct LV unloading) [8, 9]. Additionally, these studies did not utilize hemodynamic criteria to trigger LV unloading in either arm. In the third randomized trial which examined the use of VA-ECMO for shock due to acute myocardial infarction, the main drawbacks with regard to LV unloading were the lack of hemodynamic criteria (rather, pulsatility and echocardiography were utilized) as well as the low overall incidence of its use (5.8% in the early VA-ECMO arm) [17].

Results are found in Table 1. A total of 31 of 248 articles (12.5%) were found to contain suggested criteria for LVD and indications for mechanical unloading. Publication dates ranged from 2013 to 2023 [1–3, 8–10, 12, 17–39]. There were ten review articles, ten retrospective reviews five case series, three randomized trials, two guideline documents, and one editorial article. All but five articles contained clinical criteria for LVD or unloading with twenty-five describing chest radiograph evidence of pulmonary edema, and seven describing refractory ventricular arrhythmias. All but seven contained hemodynamic definitions of LVD. Seventeen included criteria regarding ALPP monitoring, fourteen included pulmonary capillary wedge pressure (PCWP) elevations, eight included elevated PAC pressures (such as pulmonary artery diastolic pressure), and three included central venous pressure (CVP) elevations. Finally, echocardiographic signs of LVD were described in all but two articles. A total of twenty articles describe smoke, echo contrast, or signs of significant stasis; seventeen articles used reduced or loss of AV opening; fifteen studies included LV size criteria, five discussed visual evidence of reduced LV contractility; three describe left ventricular outflow tract (LVOT) velocity time integral (VTI) criteria; another three describe clot in the aortic root or left ventricle; another three describe inferior vena cava diameter; and other, less frequent criteria such as significant aortic insufficiency or lung congestion index were also described. Hemodynamic criteria for LVD and the need for mechanical unloading were described in numerous studies. Of quantifiable criteria, two articles used PCWP above 18 mmHg, one above 20 mmHg, one above 30 mmHg, and three used a graded scale of PCWP. Four articles used a PAD of 25 mmHg. Three articles used an ALPP below 15 mmHg, three below 10 mmHg, and one used a graded scale.

While these criteria are helpful in the diagnosis of LVD and in establishing thresholds for mechanical LV unloading, their advantages and disadvantages must be highlighted. In what follows, we will describe the benefits and limitations of various surveillance methods for LVD.

### Review of indicators of LV distension-clinical, radiographic, hemodynamic, and echocardiographic

The most frequently used clinical indicators of LVD are significant pulmonary edema as evidenced by frothy secretions from the endotracheal tube, pulmonary hemorrhage, or ventricular arrhythmias [2, 3, 40]. These signs are often regarded as emergent indications for mechanical LV unloading and may indicate irreversible damage to cardiac muscle [1]. It is likely that the presence of clinical signs of LVD makes myocardial recovery less likely to occur. Thus, other subclinical indications of LVD should be sought for earlier detection to avoid irreversible heart failure and the subsequent need for durable left ventricular assist device placement or heart transplant.

Radiographic (chest films or chest computed tomography) indications of LVD are used to assess for the presence of pulmonary congestion [5]. These findings usually precede the clinical indicators of LVD and are often easily obtained for patients in a multitude of settings. Though helpful, these findings suggest a parenchymal abnormality and may represent a delayed finding in patients with LVD or could suggest a different pathology such as acute respiratory distress syndrome or aspiration pneumonitis.

Hemodynamic indications of LVD include elevated CVP, elevated PAC pressures (including pulmonary diastolic pressure), and PCWP [1, 2, 5]. Additionally, ALPP is an important surrogate marker for LVD and reduced contractility. In the absence of clinical or radiographic indicators of LVD, hemodynamic surveillance for LVD may identify this pathologic state before clinical and radiographic findings. Thus, many publications in the literature recommend the use of invasive hemodynamic monitoring in patients with cardiogenic shock supported by VA-ECMO [3, 5] (See Table 1).

While PAC surveillance of LVD is a very useful method of monitoring, there are drawbacks. For example, PAC positioning next to mechanical circulatory support devices (such as cannulas) can result in falsely low or high CVP readings depending on proximity to inflow or outflow ports of the drainage or return cannulas [41]. Additionally, patients with known long-standing heart failure may have elevated PAC-derived filling pressures without significant symptoms. Thus, it is difficult, if not impossible, to establish a single set of PCWP criteria to define the need for mechanical LV unloading. Finally, as an important caveat to PAC surveillance for LVD, care teams must have detailed knowledge of valvular and other cardiopulmonary abnormalities to properly interpret hemodynamic findings. For example, in patients with severe mitral stenosis, PCWP may be elevated despite normal or low left ventricular end-diastolic pressures. Additionally, in the rare case of patients with pulmonary vein stenosis, elevated PCWP does not imply increased left atrial or left ventricular pressures [42].

**Figure 1:**
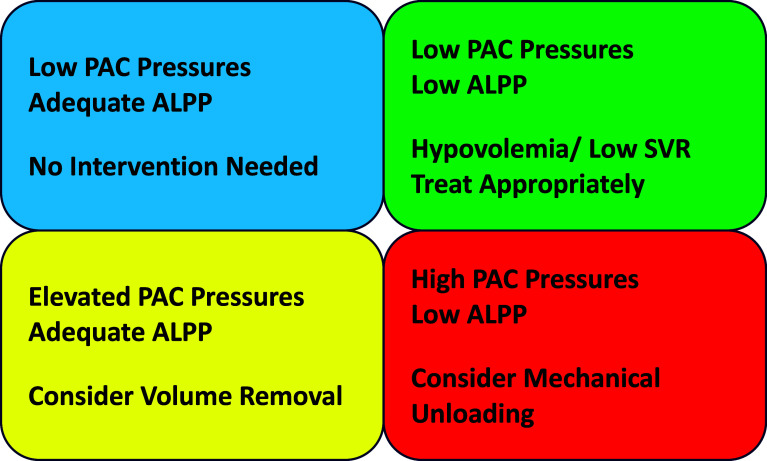
Pictorial representation of the four hemodynamic possibilities in patients supported by VA-ECMO with PAC and ALPP monitoring. Patients with adequate ALPP and low PAC-derived filling pressures do not require further unloading maneuvers. In the case of low ALPP and low PAC filling pressures, correction of hypovolemia and/or SVR will likely restore ALPP. Patients with adequate ALPP and elevated PAC filling pressures may likely require volume removal. In the case of low ALPP and elevated PAC filling pressures, the patient likely requires at a minimum non-invasive and failing that, invasive methods of LV unloading. ALPP: Arterial Line Pulse Pressure; LV: Left Ventricle; PAC: Pulmonary Artery Catheter; SVR: Systemic Vascular Resistance; VA-ECMO: Venoarterial Extracorporeal Membrane Oxygenation.

The use of ALPP monitoring for LV unloading is a basic method of surveillance for LVD. However, ALPP in patients supported by VA-ECMO can be altered for reasons other than LVD. For example, acute hypovolemia as frequently occurs with the initiation of VA-ECMO, often results in low flow through the pulmonary vasculature and thus a significant reduction in LV stroke volume. As a result, LVD can be misdiagnosed during the early stages of VA-ECMO. An acute reduction in systemic vascular resistance (SVR) can also be a cause of reduced ALPP. Other causes of acute hypovolemia such as hemorrhage or excessive diuresis may also reduce ALPP in the absence of LVD. Thus, while surveillance of ALPP is a straightforward monitoring method, it should not be solely relied upon to diagnose LVD (See Fig. 1). An important differentiation must be made between arterial pulsatility (or AV opening) and adequate LV unloading. For example, while low ALPP does suggest that the AV is opening (and aortic root thrombosis is unlikely), AV opening may not be adequate to result in sufficient ejection of blood to reduce PCWP. Thus, the presence of AV openings does not fully exclude LVD.

ECHO markers of LVD include evidence of ventricular dilation, reduced LVOT VTI, lack of or reduced AV opening, echo contrast in the left atrium or LV thrombus formation in the left atrium or left ventricle, and blood stasis or thrombosis in the aortic root [2, 5]. As with hemodynamic indicators of LVD, ECHO indicators may be misleading. For example, patients with known cardiomyopathy may appear to have LV dilation even in the presence of normal or low left heart pressures. Thus, subjective findings of LV dilation in patients supported by VA-ECMO do not always indicate the need for mechanical LV unloading. Additionally, as the LVOT VTI is a surrogate marker of stroke volume, this value can be low in the setting of hypovolemia and should not always prompt the initiation of mechanical LV unloading without further verification of the presence of LVD. The presence of echo contrast in the left atrium or LV often indicates LVD, though hypovolemia can mimic this as well. Finally, as with clinical and radiologic findings, thrombus is a late sign of LVD and while important to detect, may signify a missed opportunity for earlier intervention.

Of note, ECHO and hemodynamic markers of LVD are often directly related. For example, the presence of arterial pulsatility in the setting of VA-ECMO support implies AV opening. Additionally, the presence of LVOT VTI tracings on the echocardiogram suggests that the AV must be opening at least to some extent [2, 43].

### Non-invasive and invasive mechanical unloading strategies

Unloading strategies are undertaken to reduce the complications of LVD and are separated into non-invasive and invasive mechanical unloading strategies.

Non-invasive LV unloading strategies include the use of positive end-expiratory pressure, diuresis, afterload reduction with vasodilators, inotropes, and reducing ECMO flows to decrease afterload [5]. These strategies can be rapidly performed and are generally easily reversible. In the absence of clinical indicators of LVD, these low-risk strategies are generally attempted prior to the initiation of invasive mechanical unloading methods.

Invasive LV unloading strategies are generally undertaken in the presence of clinical indicators of LVD, or with the failure of non-invasive unloading strategies. Invasive unloading strategies include the use of intra-aortic balloon pump (IABP) counter pulsation, atrial septostomy, left atrial drainage cannula placement, left ventricular drainage cannula placement, percutaneous transvalvular micro-axial pumps such as Impella^®^ (Abiomed, Danvers, MA, USA), and percutaneous trans-aortic valve venting strategies (such as transradial catheter drainage of the LV) [2, 3, 5]. More novel techniques for LV mechanical unloading include left radial access to catheterize the LV and directly drain blood and LV apical dual lumen single cannula placement for direct LV drainage with aortic reinfusion [2, 44]. Mechanical LV unloading is the definitive step in addressing clinical and subclinical LVD, though increased risks are present [7, 45].

It is important to recognize that mechanical LV unloading strategies function in mechanistically different ways and to varying effects. For example, IABP counter pulsation functions by reducing LV afterload and improving coronary perfusion pressure but requires a sufficient degree of myocardial function to provide AV opening and LV unloading [2, 46]. Left atrial drainage functions by decreasing left atrial pressure, resulting in reductions in pulmonary congestion and decreased LV preload but will not directly facilitate the passage of blood across the AV. Direct LV drainage via a cannula through the left superior pulmonary vein or by LV apical cannulation or a percutaneous approach, where a small drainage catheter is placed via wire guidance across the aortic valve draining directly to the ECMO circuit, reduces LV volume and pressure but also does not facilitate the passage of blood across the AV [3]. Finally, percutaneous transvalvular micro-axial pump placement decreases LV pressure and propels blood out of the LV even without the presence of underlying cardiac activity [2, 5].

Venous access to perform atrial septostomy or left atrial cannula placement can damage any structure from the point of access to the left atrium with possible sequelae of bleeding, damage to major vascular structures, cardiac tamponade, and VA-ECMO circuit complications such as air entrainment. In the case of IABP or other arterial access, similar complications including limb ischemia, bleeding, aortic dissection, cardiac tamponade, and other damage to vascular or cardiac structures can occur. Other risks of additional mechanical support devices include infection, hemolysis, and renal failure.

Given the risks of invasive mechanical LV unloading strategies, establishing triggers or thresholds for their use is crucial. Triggers should also consider patient-specific factors. For example, patients with significant peripheral vascular disease may not be ideal candidates for arterial access methods of LV mechanical unloading and likely have improved risk profiles with the use of atrial septostomy or other transvenous strategies. Similarly, patients with mechanical AV replacement are not candidates for percutaneous transvalvular micro-axial pump placement. Improving patient outcomes may be facilitated by standardizing the definition of LVD and identifying indications and triggers for LV mechanical unloading.

## Summary

Although multiple large retrospective reviews suggest a survival benefit when comparing mechanical LV unloading to no unloading (without indications for unloading being known), recent prospective randomized trials have not yet supported this finding [7–9]. Additionally, a recent large retrospective review of the national inpatient sample, suggested increased mortality in VA-ECMO patients undergoing mechanical unloading with percutaneous transvalvular micro-axial pump placement [45]. A lack of uniform criteria or specific strategies for LV unloading may partially explain the negative results of prospective randomized trials. For example, of the prospective randomized clinical trials with LV unloading triggers listed, there were no PAC-derived triggers for mechanical unloading [8, 9, 17]. Additionally, to date, prospective randomized trials of LV unloading have only involved the use of left atrial cannulation for drainage and not the other methods. While left atrial cannulation and drainage can reduce pulmonary edema, it is not the most common method of mechanical LV unloading and requires specific expertise to accomplish. Additionally, simple randomization to mechanical unloading versus no unloading without a more systematic method of assessing for LVD such as PAC-derived hemodynamic data is not a sufficiently refined approach to capture the patients most likely to benefit from the mechanical LV unloading. Specifically, a low LVOT VTI or a lack of ALPP could be due to acute hypovolemia from the initiation of ECMO or low afterload from an acute reduction of SVR. Similarly, without prior knowledge of baseline cardiac function, signs of ventricular dysfunction such as low ejection fraction may not be an indication for mechanical unloading in the presence of sufficient ALPP and acceptable PAC-derived filling pressures (See Fig. 1).

Aside from the type of strategy, perhaps the most crucial future direction of research on LV mechanical unloading is elucidating a preclinical threshold (such as a combination of PAC pressures and ALPP) that results in an outcome benefit. While most clinicians with VA-ECMO experience would likely agree that signs of significant congestion such as pulmonary edema, frothy secretions, and refractory ventricular arrhythmias would be indications for mechanical LV unloading, other criteria for earlier intervention are less straightforward which highlights the need for further research. At present, it may be that employing a combination of PAC-derived hemodynamics and ALPP monitoring is the optimal, most rapid, and reliable bedside surveillance method for the early detection of LVD (See Fig. 1).

While not a systematic review, our literature search shows significant heterogeneity in definitions for the detection of LVD. It also shows a marked variation in thresholds for the initiation of LV mechanical unloading strategies. The available literature on mechanical LV unloading in patients undergoing VA-ECMO support for cardiogenic shock does not yet provide clarity on how best to proceed. At present, clinicians are faced with a dilemma regarding the risks and benefits of mechanical LV unloading strategies without a definitive reference on when and what type to initiate. It is additionally not known whether the optimal approach to mechanical LV unloading should be with early intervention (for example, at the time of VA-ECMO cannulation) or with watchful monitoring. LV unloading is a crucial part of VA-ECMO management in patients with cardiogenic shock and there is a critical need for further research on the subject. Further research on the pre-clinical diagnosis of LVD and triggers for LV unloading is of paramount importance to prevent the significant consequences of LVD while minimizing the risks of utilizing additional vascular access and procedures.

## Data Availability

This was a literature review thus, no patient data that was not already publicly available published literature was collected.
